# Effect of High-Pressure Torsion Temperatures on the Precipitation and Properties of Cu-Cr Alloy

**DOI:** 10.3390/ma17174429

**Published:** 2024-09-09

**Authors:** Yu Zhang, Depeng Shen, Guoqiang Liu, Bingtao Tang

**Affiliations:** 1 Shandong Institute of Mechanical Design and Research, Jinan 250031, China; zhangyusci@126.com (Y.Z.); liuguoqiang89@163.com (G.L.); tbtsh@hotmail.com (B.T.); 2 School of Mechanical Engineering, Qilu University of Technology (Shandong Academy of Sciences), Jinan 250353, China

**Keywords:** HPT, Cr atoms, precipitation, microhardness, electrical conductivity

## Abstract

This study examines the impact of high-pressure torsion (HPT) processing at various temperatures on the precipitation behavior of Cu-Cr alloys. The introduction of defects through HPT is observed to promote the precipitation of Cr atoms. Unlike the traditional large-scale precipitation that typically occurs around 400 °C, HPT can induce the precipitation of solute atoms even at room temperature. Furthermore, the temperature at which HPT is performed significantly influences the behavior of the precipitated phase during subsequent aging, ultimately affecting the alloy’s overall properties. At elevated temperatures (ETs) and room temperature (RT), Cr atoms tend to aggregate, forming Guinier–Preston (GP) zones or precipitates, which coarsen into incoherent precipitates after annealing. In contrast, when HPT is conducted at liquid nitrogen temperature (LNT), Cr atoms are retained in their original positions, leading to the formation of uniformly distributed, high-density small precipitates post-annealing. This phenomenon results in superior properties for HPT-LNT-treated samples, evidenced by a microhardness of 191.8 ± 3.2 HV and an electrical conductivity of 84.6 ± 1.8% IACS.

## 1. Introduction

Copper–chromium (Cu-Cr)-based alloys have garnered widespread utilization across diverse industrial sectors, including large-scale integrated circuits, transportation, aerospace, new energy vehicles, welding tools, and various other fields, due to their exceptional combination of properties such as outstanding wear resistance, high mechanical strength, and excellent electrical conductivity [1,2,3]. These alloys are particularly valued for their ability to withstand harsh operating conditions while maintaining structural integrity and performance reliability [4,5].

As typical precipitation-strengthened copper alloys, Cu-Cr systems have been extensively studied for their precipitation behavior, which plays a pivotal role in defining their mechanical and electrical properties [6,7,8]. Chbihi et al. [6] found that after annealing at 400 °C, three different kinds of precipitates coexisted in a matrix. The first type was a coherent FCC precipitate, with extinction lines parallel to the <110> Cu direction. The second type was the semi-coherent BCC precipitate, which was in the form of moiré fringes or coffee beans. The third type was the coarsened incoherent BCC precipitate or Cr particles, usually appearing elliptical or circular. Peng et al. [7] suggested that the precipitation path of Cu-0.71Cr (wt.%) alloy was as follows: supersaturated solid solution (SSS) → GP zone (FCC Cr-rich precipitates) → FCC Cr precipitates → ordered Cr precipitates → BCC Cr particles. Cheng et al. [8] studied the precipitation sequence at different temperatures. The precipitation sequence at 450 °C was SSS → FCC Cr-rich precipitates → ordered Cr-rich precipitates → ordered BCC Cr-rich precipitates. The precipitation sequence at 550 °C was SSS → ordered FCC Cr-rich precipitates → ordered BCC Cr-rich precipitates. The nature of the precipitates in Cu-Cr alloys has significant implications for their mechanical properties. The newly developed in situ TEM technique has revealed that coherent precipitates not only serve as obstacles that hinder the movement of dislocations but also facilitate the multiplication of dislocations during the deformation process [9]. Therefore, if fine FCC coherent precipitates are dominant in Cu-Cr alloys, the dislocation can cut and pass through the precipitates with shear, resulting in a significant increase in the alloys’ yield strengths, with a minimal negative impact on ductility [10,11]. However, if most of them evolve into incoherent BCC precipitates, the interaction between precipitates and dislocation will change to a bypass mechanism, and the reinforcement caused by precipitates is thereby weakened [12,13]. In addition, if discontinuous precipitation occurs, the coarsened incoherent precipitates will segregate and come to be located at grain boundaries, leaving the alloy prone to instability fracture under plastic strain [14,15].

Thermo-mechanical treatments are typically applied to precipitation-strengthened Cu–Cr–Zr alloys to attain high strength and good electrical conductivity, which could improve the mechanical properties through grain-refining strengthening [14,16,17,18,19]. For example, Fu et al. [16] conducted the rolling of a Cu-0.98wt%Cr-0.057wt%Zr alloy at 300 °C, resulting in the formation of a high-density, small-sized precipitated phase within the matrix. This suggests that plastic deformation at a moderate temperature can induce the dissolution of solid-soluble atoms. At this stage, the tensile strength reached 657.7 MPa. However, the electrical conductivity was approximately 40% IACS, indicating that the dissolution of solid-soluble atoms was incomplete, and the peak aging state had not been achieved. Caldatto Dalan et al. [17] studied the effect of equal-channel angular pressing (ECAP) processing at room temperature and 300 °C on the distribution of the second-phase particles and its influence on the hardness and electrical conductivity of a commercial Cu-0.81Cr-0.07Zr alloy. The results showed that the area fraction of coarse Cr-rich particles decreased after ECAP processing, which was attributed to the Cr dissolution induced by severe plastic deformation. And annealing treatment promoted an additional hardening effect and the complete recuperation of the electrical conductivity, caused by the re-precipitation of the partially dissolved particles. Li et al. [18] found that after two-step cryo-rolling and aging treatment, a better combination of the tensile strength (648 MPa) and electrical conductivity (79.80% IACS) can be obtained. Guo et al. [19] found that when the Cu-0.6Cr alloy was extruded by equal-channel angular pressing (ECAP) under liquid nitrogen cooling, the amounts of micro/nanoprecipitates increased after annealing treatment. Their findings indicated that deformation at lower temperatures may promote the formation of Cr precipitates. The synergistic effect of precipitates and the formation of {111} <112> and {111} <110> textures were beneficial for improving the properties, and the tensile strength, hardness, and electrical conductivity reached 555.0 MPa, HV 167.3, and 84% IACS, respectively. 

In light of these findings, it is evident that the deformation temperature plays a crucial role in dictating the precipitation behavior and consequently affects the mechanical properties of Cu-Cr-based alloys. However, the precise mechanisms underlying these effects require further elucidation. As is known, high-pressure torsion (HPT) is a prominent severe plastic deformation (SPD) technique extensively employed to achieve significant microstructural refinement and enhance the mechanical and physical properties of materials [20]. This method is particularly noted for inducing extreme grain refinement and generating a high density of lattice defects. During the HPT process, a disk-shaped specimen is positioned between two anvils and subjected to an exceptionally high compressive load, typically reaching several gigapascals. Concurrently, one anvil undergoes rotation relative to the other, imparting a torsional strain on the sample. The synergistic effect of the applied high pressure and shear deformation leads to profound plastic deformation, resulting in the fragmentation of grains and the introduction of a substantial density of dislocations [20]. This extreme deformation markedly transforms the microstructure, facilitating phenomena such as grain boundary sliding, lattice distortion, and phase transformations that are crucial for defining the material’s mechanical properties [21]. This capability enables a systematic exploration of the effects of deformation temperature on microstructural evolution, providing valuable insights into the underlying mechanisms that dictate the material’s properties.

In this study, a Cu-0.86 wt.% Cr alloy was subjected to high-pressure torsion (HPT) at varying temperatures: liquid nitrogen (HPT-LNT), room temperature (HPT-RT), and elevated temperatures (HPT-ETs). The effects of deformation temperature on the precipitation behavior, as well as the mechanical and electrical properties of the alloy, were systematically investigated.

## 2. Experimental Section

The Cu-0.86 wt% Cr alloy was synthesized by utilizing a vacuum-induction melting furnace. The precise control of the melting environment ensured the minimization of contamination and oxidation, yielding a high-purity alloy. Post-melting, the resultant ingot underwent a rigorous solution treatment (ST) at 1000 °C for a duration of 1 h, immediately followed by rapid water quenching. Then, the alloy ingot was sectioned into disk-shaped specimens, each with a diameter of 10 mm and a thickness of 1 mm. These specimens were then subjected to HPT. HPT processing was conducted at various temperatures to investigate the temperature-dependent behavior of the alloy. The temperatures selected for the study included liquid nitrogen cooling temperature (LNT), room temperature (RT), and elevated temperature (~200 °C) (ET). Each specimen was processed at a rotational speed of 1 rpm under a constant pressure of 1.5 GPa, applied using a constrained anvil setup. The application of HPT under these meticulously controlled conditions aimed to systematically explore the microstructural evolution, mechanical property enhancement, and thermal stability of the Cu-0.86 wt% Cr alloy. The thermal response during heating was analyzed using differential thermal calorimetry (DSC). Samples weighing 90 mg were heated to 515 °C in an argon atmosphere. Phase identification was conducted via X-ray diffraction (XRD). Microstructural observations and electron diffraction were performed with a TALOS F200X transmission electron microscope (TEM) at 200 kV. TEM samples were prepared by mechanically thinning the material to approximately 100 µm, followed by ion milling until the sample was electron-transparent. The final thickness was typically less than 100 nm, ensuring suitability for high-resolution TEM analysis. Electrical conductivity measurements were taken using an intelligent eddy-current low-resistance tester, with the average value and standard deviation calculated from at least 10 measurements. Mechanical properties were assessed through Vickers microhardness testing.

## 3. Results and Discussion

**Figure 1:**
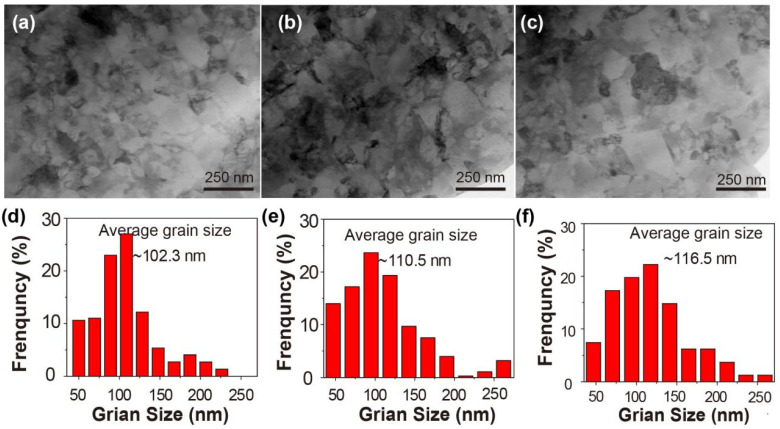
The TEM micrographs and grain size distribution of the HPT-processed samples of (**a**,**d**) HPT-LNT-20; (**b**,**e**) HPT-RT-20; and (**c**,**f**) HPT-ET-20.

The microstructures and grain size distribution of the HPT-processed samples are shown in Figure 1. The grain size is significantly refined, leading to the formation of an ultra-fine crystalline structure. The grain size distributions indicate that the average grain size increases as the processing temperature rises. Specifically, the average grain sizes was approximately 102.3 nm for the HPT-LNT-20 sample, 110.5 nm for the HPT-RT-20 sample, and 116.5 nm for the HPT-ET-20 sample. The increase in grain size with higher processing temperatures is a direct consequence of enhanced atomic mobility, accelerated re-crystallization, and reduced defect density [22,23]. 

**Figure 2:**
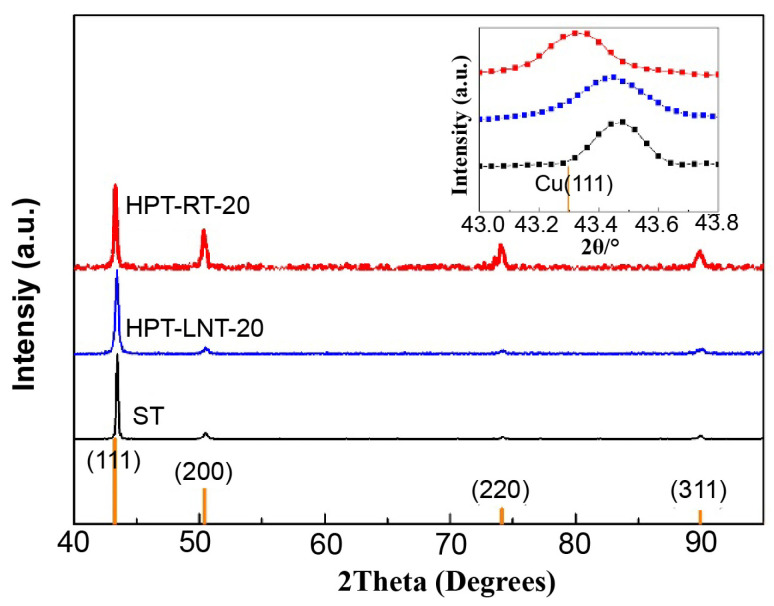
XRD pattern of the as-solute coarse-grained Cu-Cr alloys as well as HPT-processed specimens of HPT-LNT-20 and HPT-R-20.

Figure 2 presents the X-ray diffraction (XRD) patterns of the solution-treated coarse-grained Cu-Cr alloy alongside specimens subjected to high-pressure torsion (HPT) at cryogenic temperature (HPT-LNT) and ambient temperature (HPT-RT) for 20 rotations. The analysis reveals that the diffraction peaks in HPT-processed samples exhibit significant broadening in comparison to those in the solution-treated alloy, a consequence of increased lattice defects, including dislocations, grain boundaries, and the resultant grain refinement. Such broadening, quantified by the full width at half maximum (FWHM) values, underscores the microstructural evolution induced by severe plastic deformation. In addition to peak broadening, the diffraction peak positions for both the solution-treated and HPT-processed samples show discernible shifts when compared to the standard Cu peaks, indicative of changes in lattice constants relative to pure Cu. These shifts were systematically quantified by determining the lattice constants through the least squares method [24], applied to the Cu(111), Cu(200), Cu(220), and Cu(311) reflections. The data show that the lattice parameter of the solution-treated Cu-Cr alloy is approximately 3.60325 Å, which is slightly smaller than the lattice parameter of pure Cu, 3.6149 Å. This contraction is attributed to the incorporation of Cr atoms into the Cu matrix, which have a smaller atomic radius (0.1249 nm) than Cu atoms (0.1278 nm) [25], thereby reducing the overall lattice spacing. Post HPT processing, distinct behaviors are observed depending on the processing temperature. The diffraction peak positions for the cryogenically processed sample (HPT-LNT-20) remain largely unchanged, suggesting minimal lattice relaxation or recovery. Conversely, the ambient-temperature-processed sample (HPT-RT-20) exhibits a noticeable shift towards lower angles, indicating an expansion in lattice parameters. This behavior suggests that room-temperature HPT facilitates the precipitation of Cr atoms, as corroborated by earlier studies [26], which demonstrated that the lattice parameter of Cu decreases progressively with increasing Cr solubility. Thus, the observed lattice expansion in the HPT-RT sample may result from the partial dissolution of Cr atoms back into the matrix, alleviating internal stresses and causing the peak shift. These findings provide critical insights into the solute behavior and microstructural stability of Cu-Cr alloys under extreme deformation conditions, underscoring the temperature-dependent mechanisms governing lattice parameter evolution and Cr atom mobility during HPT.

**Figure 3:**
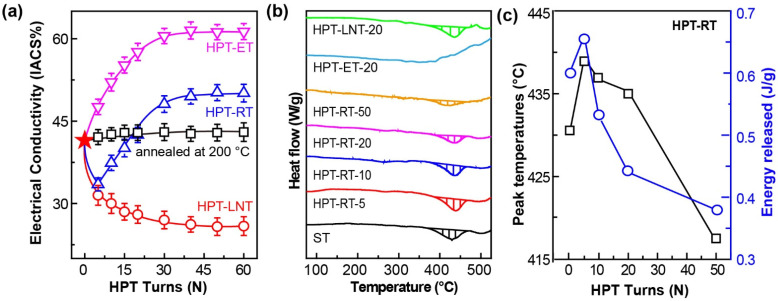
(**a**) The EC of HPT-processed samples varies with HPT turns; (**b**) DSC curves. (**c**) The peak position and energy release of the exothermic peak vary with HPT turns at RT. The red asterisk represents the electrical conductivity of the ST sample, while the black square depicts the change in electrical conductivity of the ST sample as a function of aging time at 200 °C. The red hollow circles, blue triangles, and pink inverted triangles represent the variation in electrical conductivity of the ST sample with the number of processing revolutions at LNT, RT, and ET, respectively.

Figure 3a highlights the variations in EC for HPT-processed samples with differing numbers of turns. Notably, the EC of the HPT-LNT samples exhibits a gradual decline, attributed to the proliferation of dislocations and grain boundaries introduced by the HPT process. This decrease aligns with established understanding that an increased dislocation density and grain boundary areas impede electron flow, thus reducing conductivity [27,28]. In contrast, the EC of solution-treated (ST) samples remains relatively stable when annealed at 200 °C. This stability is consistent with the absence of significant large-scale precipitation, which typically occurs around 400 °C [29]. Interestingly, HPT processing at room temperature (RT) and 200 °C results in increased EC to approximately 50% and 61% International Annealed Copper Standard (IACS), respectively. It is well known that the conductivity is almost linear with the atomic residue of the solute, and the less the residue, the higher the conductivity [30,31]. This increase suggests that the HPT-induced dislocations and grain boundaries facilitate Cr precipitation even at room temperature. Figure 3b,c provide DSC curves, revealing an exothermic peak associated with Cr precipitation. The observed peak temperature and energy release amount (ERA) initially increase with the number of HPT turns but subsequently decrease. This behavior indicates that while HPT initially promotes Cr solution, continued deformation leads to a partial precipitation of Cr atoms, reducing the content of retained solution Cr. The diminishing ERA and the eventual disappearance of the peak in HPT-ET samples suggest that extensive HPT at an elevated temperature results in nearly complete precipitation of Cr atoms from the solid solution. This finding is in line with previous studies demonstrating that dislocation and grain-boundary-induced nucleation can significantly accelerate precipitation kinetics at room and elevated temperatures.

**Figure 4:**
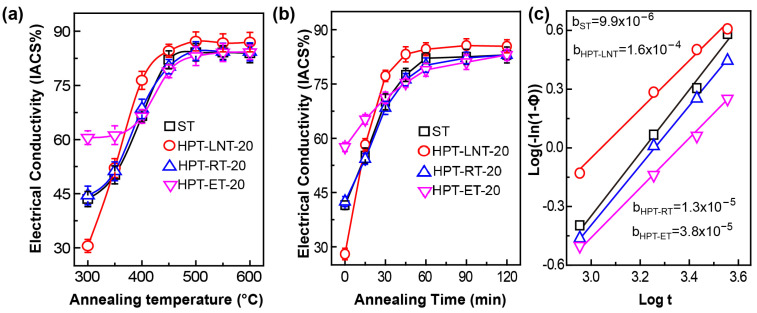
(**a**) The EC of HPT-processed samples varies with temperature. (**b**) The EC of HPT-processed samples varies with annealed time at 450 °C. (**c**) The fitted straight line by the logarithmic Avrami equation.

Figure 4a,b illustrate the variation in electrical conductivity (EC) of HPT-processed samples with 20 turns, as a function of annealing temperature and time. The EC serves as an indicator of the relative volume fraction of precipitates (V_P_), as expressed in Equation (1) [32].

(1) $$\sigma =\sigma_{0}-V_{P}$$

where σ is the EC with a different time of annealing treatment. σ_0_ represents the electrical conductivity of the solution-treated (ST) sample. This parameter was determined by measuring the electrical resistance of the original coarse-grained sample using a digital micrometer, and then calculating the conductivity based on Ohm’s law. According to Figure 3b, a straight line can be fitted by combining the logarithmic Avrami equation (Equation (2)) [14], as shown in Figure 3c.

(2) $$\log(1-\frac{\sigma }{\sigma_{0}})=bt^{n}$$

where t denotes the annealing time; b is the Avrami rate constant; and n is the Avrami exponent. The Avrami coefficient b is a function of both nucleation rate and growth rate and is related to the geometry of the growing nuclei [33,34]. A higher b value indicates a faster precipitation rate [34].

Notably, the b values for HPT-processed samples are higher than those for ST samples, suggesting that the deformation induced by HPT enhances the precipitation rate. Among the HPT-processed samples, the HPT-LNT-20 samples exhibit the highest b values, indicating the most rapid precipitation rates. These observations are consistent with previous studies on the effect of severe plastic deformation on precipitation kinetics. For instance, it has been reported that severe plastic deformation can significantly accelerate the diffusion processes, leading to enhanced nucleation and growth rates of precipitates [35,36]. This is particularly evident in the HPT-processed samples, where the increased dislocation density and grain boundary area provide abundant nucleation sites for precipitates [37]. Consequently, the precipitation process in HPT-processed samples is more efficient compared to ST samples.

**Figure 5:**
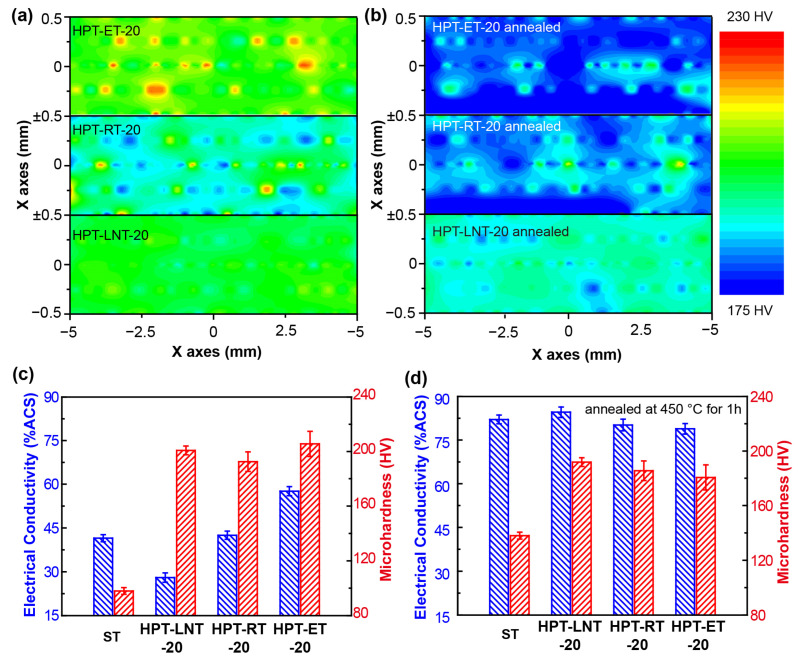
The microhardness distribution of (**a**) HPT-processed samples (**b**) and annealed HPT-processed samples. The average microhardness and EC of (**c**) HPT-processed samples and (**d**) the samples annealed at 450 °C for 1 h.

Figure 5a,b depict the microhardness distribution along the X-axis of the HPT-processed alloys with 20 turns, both before and after annealing. Figure 5c,d show the average hardness EC of these samples. The microhardness of HPT-RT-20 was the lowest, whereas HPT-ET-20 exhibited the highest microhardness. Notably, compared to HPT-LNT-20, the microhardness distribution in HPT-RT-20 and HPT-ET-20 was less uniform. This suggests that high-density precipitates formed locally when deformation occurred at room temperature (RT) and elevated temperature (ET). Post annealing treatment, the microhardness (191.8 ± 3.2 HV) and EC (84.6 ± 1.8% IACS) of HPT-LNT-20 surpassed those of both HPT-RT-20 and HPT-ET-20. The non-uniform microhardness distribution in HPT-RT-20 and HPT-ET-20 can be attributed to the localized generation of high-density precipitates during deformation. This localized precipitation is likely due to the heterogeneous nature of the deformation process at RT and ET, which creates regions with varying dislocation densities and grain boundary characteristics. As a result, precipitates preferentially form in areas with higher defect concentrations, leading to a non-uniform microhardness distribution. In contrast, the HPT-LNT-20 samples exhibited a more uniform microhardness distribution and superior mechanical and electrical properties post-annealing. The superior properties of HPT-LNT-20 can be explained by the homogeneous deformation and enhanced diffusion processes at low temperatures, which promote a more uniform distribution of precipitates. Additionally, low-temperature deformation can lead to a higher density of dislocations and vacancies, providing more nucleation sites for precipitates and resulting in a finer and more uniform precipitation distribution [38,39]. Furthermore, the annealing treatment plays a crucial role in enhancing the properties of HPT-processed samples. Annealing allows for recovery and recrystallization processes, reducing the dislocation density and promoting precipitation. This results in improved electrical conductivity. The superior microhardness and EC of HPT-LNT-20 samples underscore the importance of the processing temperature and subsequent annealing treatment in optimizing the properties of HPT-processed alloys. 

**Figure 6:**
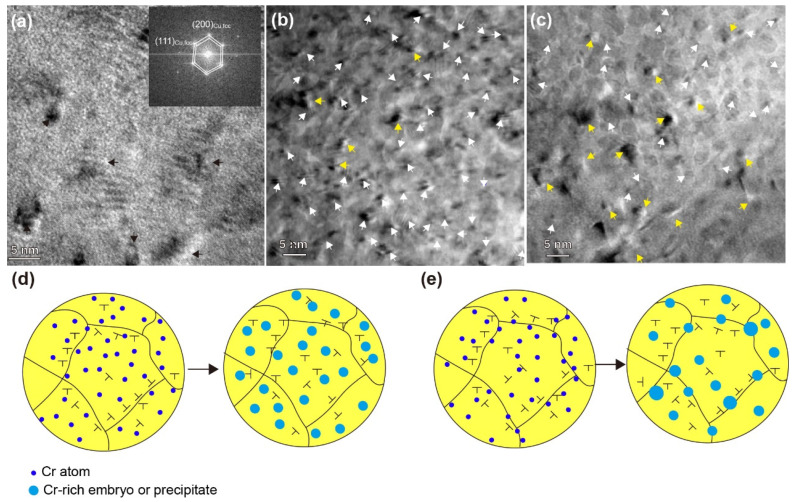
TEM micrographs of the samples of (**a**) HPT-LNT-20; (**b**) HPT-RT-20; (**c**) and HPT-ET-20; Schematic of precipitation behavior of the samples of (**d**) HPT-LNT-20 (**e**) and HPT-RT-20. The yellow and white arrows indicate the coarsened incoherent precipitates and the fine coherent Cr-rich nanoprecipitates, respectively.

To elucidate the above phenomenon, further characterization was performed, as illustrated in Figure 6. Interestingly, spherical or moiré fringe contrast were found in the HPT-RT sample, as indicated by the black arrows. According to SADE analysis, this is an FCC coherent precipitate (Figure 6a). This further confirms that the dislocations or grain boundaries continuously introduced by HPT can promote the occurred of pre-precipitation without any annealing treatment. In addition, a high density of FCC coherent precipitates (white arrows) was found in the annealed HPT-LNT-20 sample (Figure 6b). Meanwhile, in the annealed HPT-RT-20 sample, the density of precipitates was much lower, and more coarsened incoherent precipitates were observed (yellow arrows), as shown in Figure 6c. Since the reinforced particles were dominated by FCC coherent precipitates with a smaller size and higher density, the overall performance of the annealed HPT-LNT-20 was better than those of other samples. A schematic was created to explain the above phenomena, as shown in Figure 6d,e. Due to the extremely low diffusion at LNT, the Cr atoms will be locked in the original location during HPT, and they will form high-density precipitates with a uniform distribution after annealing treatment (Figure 6d). When HPT was performed at RT or ET, defects like dislocations and grain boundaries were continuous introduced, which could act as preferential nucleation sites. As a result, Cr atoms were prompted to move and aggregate towards these sites and formed GP zones, and they further evolved into coarsened incoherent precipitates after annealing treatment. At this point, some Cr-depleted regions formed, and thus the precipitation kinetics and the density of precipitates decreased when subjected to annealing treatment (Figure 6e). 

**Figure 7:**
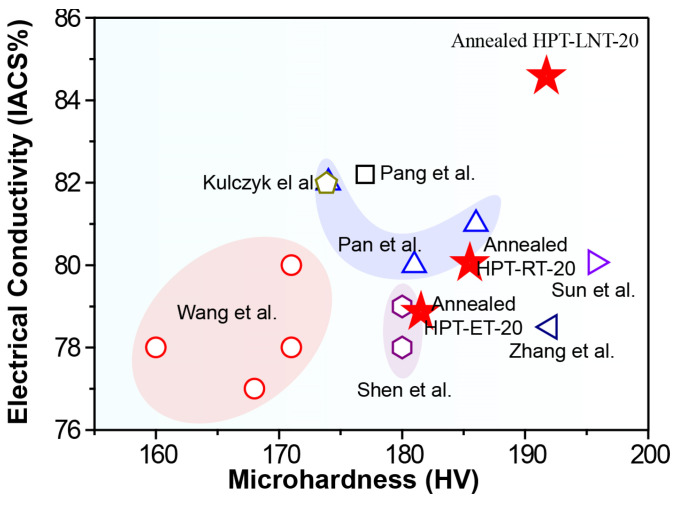
Comparison of electrical conductivity and hardness of Cu-Cr alloy after thermo-mechanical treatment as reported in previous studies with the results obtained in this work [5,40,41,42,43,44]. Different shapes, including hollow circles, hexagons, and triangles, represent the performance of previously published research results, while the red five-pointed star denotes the performance achieved in this study.

Figure 7 provides a comprehensive comparison of the electrical conductivity and hardness of Cu-Cr alloys subjected to various thermo-mechanical treatments, as documented in prior studies, juxtaposed with the results from this investigation (represented by the red pentagon symbol). The data distinctly highlight the superior performance of the HPT-LNT-20 treatment, which achieves an exceptional combination of mechanical strength and electrical conductivity. The pronounced synergy observed between these properties illustrates the profound impact of the HPT-LNT-20 process in refining the microstructure of Cu-Cr alloys. This process effectively promotes grain refinement, enhances precipitation behavior, and minimizes lattice defects, leading to a significant improvement in material performance. The findings underscore the potential of HPT-LNT-20 as a transformative approach in the design of Cu-Cr alloys, offering a robust pathway for developing materials with optimized properties for demanding technological applications.

## 4. Conclusions

(1) High-pressure torsion (HPT) processing at room temperature (RT) and elevated temperatures (ETs) leads to the formation of defects, which in turn promote the precipitation of chromium (Cr) atoms. Notably, this precipitation can occur even at room temperature, as the Cr atoms tend to aggregate and form Guinier–Preston (GP) zones during HPT. These zones further evolve into coarsened, incoherent precipitates following annealing treatment.

(2) During HPT at LNT, Cr atoms may become locked in their original locations, resulting in higher-density and more uniformly distributed precipitates after annealing. Consequently, annealed samples exhibit a smaller size and higher density of nanoprecipitates in HPT-LNT compared to those treated at RT and ET, which contributes to the superior performance of these samples. Specifically, the HPT-LNT samples demonstrate a notable microhardness of 191.8 ± 3.2 HV and an electrical conductivity of 84.6 ± 1.8% IACS.

## Data Availability

The original contributions presented in the study are included in the article, further inquiries can be directed to the corresponding author.
